# Pathogenicity of Multidrug-Resistant *Salmonella typhimurium* Isolated from Ducks

**DOI:** 10.3390/microorganisms12071359

**Published:** 2024-07-02

**Authors:** Yulin Xu, Zhitong Yu, Shaopeng Wu, Mengze Song, Lulu Cui, Shuhong Sun, Jiaqiang Wu

**Affiliations:** 1 Shandong Key Laboratory of Animal Disease Control and Breeding, Institute of Animal Science and Veterinary Medicine, Shandong Academy of Agricultural Sciences, Jinan 250100, China; ylxu15650096726@163.com; 2 Key Laboratory of Livestock and Poultry Multi-Omics of MARA, Jinan 250100, China; 3 Shandong Provincial Key Laboratory of Animal Biotechnology and Disease Control and Prevention, College of Animal Science and Veterinary Medicine, Shandong Agricultural University, Tai’an 271000, China; 13147904840@163.com (Z.Y.); wsp@sdau.edu.cn (S.W.); songmz@sdau.edu.cn (M.S.); 2021010090@sdau.edu.cn (L.C.)

**Keywords:** *Salmonella typhimurium*, animal experiment, multidrug-resistant, pathogenicity

## Abstract

*Salmonella typhimurium* (*S. typhimurium*) is one of the most common *Salmonella* serotypes in epidemiological surveys of poultry farms in recent years. It causes growth retardation, mortality, and significant economic losses. The extensive use of antibiotics has led to the emergence of multi-drug resistance (MDR) in Salmonella, which has become a significant global problem and long-term challenge. In this study, we investigated the prevalence and features of *S. typhimurium* strains in duck embryos and cloacal swabs from large-scale duck farms in Shandong, China, including drug resistance and virulence genes and the pathogenicity of an *S. typhimurium* strain by animal experiment. The results demonstrated that a total of 8 *S. typhimurium* strains were isolated from 13,621 samples. The drug resistance results showed that three of the eight *S. typhimurium* strains were MDR with the dominant resistance profile of CTX-DX-CTR-TE-AMX-AMP-CAZ. In particular, the virulence genes *invA*, *hilA*, *pefA*, *rck,* and *sefA* showed high positive rates. Based on the analysis of the biological characteristics of bacterial biofilm formation and mobility, a strain of *S. typhimurium* with the strongest biofilm formation ability, designated 22SD07, was selected for animal infection experiments with broiler ducklings. The results of animal experiments demonstrated that infection with 22SD07 reduced body weight and bursa index but increased heart and liver indexes compared to the control group. Histological examination revealed desquamation of the intestinal villous epithelium, the presence of large aggregates of lymphocytes, and a decrease in goblet cells following infection. Furthermore, the expression of IL-10 was significantly increased in the liver at 3 dpi, while TNF-α was significantly increased in the spleen at 7 dpi. The above results indicate that *S. typhimurium* may pose a potential threat to human health through the food chain. This helps us to understand the frequency and characteristics of *S. typhimurium* in duck farms and emphasizes the urgent need to strengthen and implement effective continuous monitoring to control its infection and transmission.

## 1. Introduction

Salmonella is one of the most important zoonotic pathogens, and the widespread use of antibiotics in the poultry industry has led to the emergence of MDR Salmonella [1,2]. The indiscriminate and unregulated use of antibiotics has led to an increase in the prevalence of MDR Salmonella in animals and humans, and MDR Salmonella can be transmitted to humans through animal products or the environment, posing significant public health and food safety concerns [3,4]. It can be transmitted in the environment through animal feces [5] and to humans through various processes used in the production of food of animal origin [6], posing a serious threat to food safety and public health [7]. *S. typhimurium* is MDR Salmonella with a high detection rate in epidemiological surveys of poultry farms in recent years—which not only causes chick mortality but can also colonize adult chickens for a long time, leading to asymptomatic persistent infections—and is also one of the most important serotypes of salmonella transmitted from animals to humans in most parts of the world [8,9]. The prevalence of Salmonella isolated from fresh duck meat in the southern retail market of China has been reported. The results show that a high level of Salmonella contamination is detected in duck meat (151/365, 41.4%). Among them, *S. typhimurium* accounted for 6/151 [10]. Kang et al. found that *S. typhimurium* was distributed in various production stages in the duck production chain [11]. Zhao conducted an epidemiological investigation on Salmonella isolated from several duck farms in Shandong Province and its surrounding areas, among which Kottbus and *S. typhimurium* were the dominant serotypes [12]. The Import and Export Food Safety Inspection and Monitoring Programme has adopted a zero-tolerance policy for Salmonella in food safety inspection and monitoring programs. With increasing awareness of the evolution of antimicrobial resistance (AMR) in Salmonella, longitudinal surveillance programs have been initiated by governments and researchers, providing valuable epidemiological data for risk assessment and medication guidelines [13].

Biofilms are dynamic communities of heterogeneous microorganisms that transition from a free-swimming planktonic state to a sessile state embedded in the extracellular matrix [14,15]. Several AMR phenotypes and genes, particularly those conferring resistance to fluoroquinolones, have been reported to be associated with biofilm formation [16]. In addition, biofilm formation is closely associated with pathogenicity, and reports on the relationship between AMR pathogenicity and biofilms are inconsistent, suggesting that the true relationship is unclear [17].

Virulence factors enable bacteria to enter, adhere to, and replicate in host cells. Virulence factors also enable bacteria to overcome host defenses and cause disease [18]. Salmonella has several known virulence factors present in Salmonella pathogenicity islands (SPIs), phages, bacterial clusters, and plasmids. Most virulence genes involved in pathogenesis are located in SPI-1 and SPI-2 pathogenicity islands in the Salmonella genome [18]. Duck meat is a popular meat product, and China is the world’s largest producer and consumer of farmed waterfowl [19]. Our previous findings confirmed the high isolation rate of Salmonella from dead embryos and meconium of newly hatched chicks [20] and that infection with *S. typhimurium* alters the intestinal microbiota by increasing the abundance of *Rumatococcaceae* and *Serratiaceae* [21]. In this study, we analyzed the epidemiology, drug resistance characteristics, and virulence genes of *S. typhimurium* in 239 large-scale duck farms in selected areas of Shandong Province. Furthermore, the *S. typhimurium* strain with strong biofilm formation ability was tested in animal experiments to investigate its pathogenicity to ducklings.

## 2. Materials and Methods

### 2.1. Main Reagents

Buffered peptone water (BPW), tetrathionate broth (TTB-enriched broth), selenocysteine (SC)-enriched broth, and xylose-lysine deoxycholate (XLD) were purchased from HaiBo BioTech Ltd. (Qingdao, China). Antibiotic susceptibility test plates were purchased from Hangzhou Microbial Reagent Co., Ltd. (Hangzhou, China). Diagnositic Sera Kit for Salmonella was purchased from Ningbo Tianrun Bio-pharmaceutical Co., Ltd. (Ningbo, China).

### 2.2. Sampling Strategy and Isolation and Identification of Salmonella

Using the method described previously [20], 13,621 samples (361 duck embryos and 13,260 cloacal swabs) for Salmonella isolation and identification were collected from 239 duck farms in Shandong Province between September 2022 and November 2023, and the samples were stored in a low-temperature environment and transferred to the laboratory. In short, each sample was added to 4.5 mL of BPW and then incubated at 37 °C for 12 h for pre-enrichment. Approximately 0.5 mL of pre-enriched culture was inoculated into 4.5 mL of TTB and SC, respectively. Cultures of each TTB and SC broth were inoculated on an XLD Agar base and incubated at 37 °C for 48 h [22]. The confirmation of smooth and round colonies without a black center or large with a black center was achieved by polymerase chain reaction (PCR) assays with primers designed for the *FimW* gene [20].

### 2.3. Drug Susceptibility Test

All Salmonella strains were tested for resistance to 15 antimicrobial drugs by the broth-diffusion method according to the Clinical and Laboratory Standards Institute (CLSI) [23]. The antibiotics used were ceftriaxone (CTR, 30 μg), ceftazidime (CAZ, 30 μg), ampicillin (AMP, 10 μg), amoxicillin (AMX, 20 μg), meropenem (MEM, 10 μg), tetracycline (TE, 30 μg), doxycycline (DOX, 30 μg), amikacin (AMK, 30 μg), enrofloxacin (ENR, 15 μg), ofloxacin (OFX, 30 μg), azithromycin (AZI, 30 μg), sulfamethoxazole (SXT, 25 μg), and sulfamonomethoxine (SMM, 30 μg). Polymyxin B (PB) susceptibilities were determined using the microbroth dilution assay [24]. MDR was defined as resistance to three or more classes of antimicrobial agents.

### 2.4. Detection of Salmonella Serotype

Salmonella serotype was detected using the Diagnostic Sera Kit for Salmonella (Ningbo Tianrun Bio-pharmaceutical Co., Ltd., Ningbo, China) according to the instructions.

### 2.5. Detection of Virulence Genes

Genomic DNA was extracted with the Genomic DNA Purification Kit (Tiangen Biotech, Beijing, China), and DNA templates were stored at −20 °C before use [25]. Eight virulence genes (*invA*, *hilA*, *sipC*, *sopE*, *spvC*, *ssaR*, *ssrA*, and *stnP1*) were detected in all isolates, as previously described [26]. Ten PCR products were randomly selected and sequenced for each gene, and the sequences were then analyzed by sequence comparison using the NCBI database to verify the accuracy of the sequences.

### 2.6. Biofilm Formation Assay

AS previously described [27], the overnight cultures of *S. typhimurium* strain were diluted to an OD_600_ of 1000-fold in 3 mL of fresh LB broth in polystyrene tubes at 37 °C with shaking for 12 h. After incubation, the growth medium was decanted, and the tubes were washed three times with sterile PBS buffer and air-dried. Biofilms were quantified using a crystal violet assay by adding 250 μL of 0.05% crystal violet solution to each well. Plates were incubated for 15 min at room temperature and rinsed with distilled water. The crystal violet was dissolved in 200 μL of 95% ethanol, and biofilm formation was analyzed at OD_570_. Each strain of *S. typhimurium* was tested in three independent replicates.

### 2.7. Motility Assay

The freshly cultured bacterial solution was adjusted to an OD_600_ of 0.1, and 1 μL was pipetted into the semi-solid LB solid medium at a depth of approximately 0.5 mm using a 10 μL of pipette and then incubated in a constant temperature incubator at 37 °C for 4–24 h. The growth of the strain was observed, and the diameter of the strain was measured [28]. Each strain of *S. typhimurium* was tested in three independent replicates.

### 2.8. Animal Experiment

Firstly, the infection model of broiler ducklings was constructed to obtain the LD_50_ of the leg muscle injection dose as 3.1 × 10^8^ CFU. To further explore the pathogenicity of *S. typhimurium* to broiler ducklings, 24 one-day-old Cherry Valley ducklings were purchased from Ningyang Juxiang Breeding Co., Ltd., Tai’an City, Shandong Province, China. The ducklings were randomly divided into 2 groups and placed in a clean and tidy environment at 32 °C with unrestricted access to food and drinking water. *S. typhimurium* strain 22SD07 was cultured to logarithmic growth stage, washed with PBS, and diluted to 10^8^ CFU/mL. After 3 days, the challenge doses were 10^8^ CFU/mL each (≈0.32 LD_50_) of the 100 μL bacterial solution for the intraperitoneal injection group, and controls were injected with the same dose of saline. The ducklings were weighed and recorded at 1, 3, and 7 dpi, and the heart, liver, spleen, cecum, and bursa were collected at 3 and 7 dpi from 6 ducklings to calculate the visceral tissue indexes, part of which were stored in the −80 °C and paraformaldehyde for subsequent pathological sectioning. Heart, liver, spleen, thymus, and bursa were collected for the determination of Salmonella counts. The immune organ index was calculated as follows: immune organ index (g/kg) = immune organ weight (g)/live weight of chicks (kg). Tissue samples were weighed and homogenized in PBS, and serial dilutions of the homogenates were plated onto XLD plates (37 °C, 16 h) for bacterial enumeration.

### 2.9. Real-Time Quantitative PCR

The expression of cytokines, including interleukin-4 (IL-4), IL-6, IL-10, IFN-γ, and tumor necrosis factor (TNF-α), was analyzed by qPCR. RNA from liver, spleen, and cecum was extracted using TRIzol reagent (Thermo Fisher Scientific, Waltham, MA, USA), and cDNA synthesis was performed using 1 μg of RNA template via the Transcriber First Strand cDNA Synthesis Kit (Roche, Basel, Switzerland). Appropriate primer sets were used for qPCR (Table 1) on the ABI 7500 Detection System (Applied Biosystems, Carlsbad, CA, USA) using 18S as an internal reference. The PCR procedure consisted of 95 °C for 30 s, 40 cycles of 95 °C for 5 s, and then 60 °C for 30 s.

### 2.10. Data Analysis

The results were analyzed using GraphPad Prism (Version 8.0.1) and expressed as mean ± SD. Student’s *t*-test was used to assess the differences between two groups. Results were considered significant when * *p* < 0.05, ** *p* < 0.01, and *** *p* < 0.001.

## 3. Results

### 3.1. Isolation, Serotype, and Antibiotic Resistance of Salmonella Strains

A total of 44 Salmonella strains were isolated from 361 dead embryos and 13,260 cloacal swabs with a positivity rate of 0.32% (44/13,621). Eight of these strains were *S. typhimurium*, designated 22SD01-22SD08. The results of the antimicrobial susceptibility analysis of 8 *S. typhimurium* varied in resistance to 14 antibiotics, as shown in Table 2. MDR strains accounted for 37.5% (3/8). Among them, the highest rate of resistance to DOX and AK reached 75% (6/8) and 62.5% (5/8), respectively (Table 3). Notably, SD2207 had a resistance profile of CTX-DX-CTR-TE-AMX-AMP-CAZ.

### 3.2. Virulence Genes of S. typhimurium

The identification of eight virulence genes in *S. typhimurium* strains is shown in Table 1. Apart from 22SD04, which carried six virulence genes (*invA*, *sipC*, *sipA*, *ssaR*, *ssrA,* and *stnp1*), the other seven strains carried *invA*, *sipC*, *sipA*, *ssaR*, *ssrA*, *stnp1,* and *hilA*.

### 3.3. Analysis of the Biofilm Formation Ability and Motility of S. typhimurium

**Figure 1:**
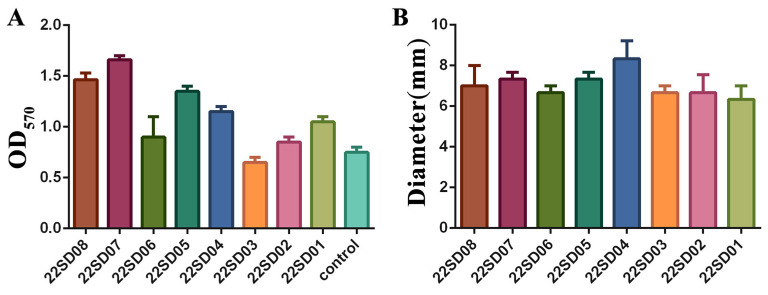
Determination of biofilm formation ability and motility of strain 22SD07. (**A**) Comparison of biofilm formation ability of eight strains of *S. typhimurium*. (**B**) Comparison of the motility ring diameter of eight strains of *S. typhimurium*.

The biofilm formation ability of different strains of *S. typhimurium* was first assessed. Different clinical strains showed different abilities to form biofilms, with strain 22SD07 showing the most robust biofilm-forming capacity (Figure 1A). In addition, the results of the motility assay are shown in Figure 1B, where the motility ring of strains on the semi-solid flat dish showed no significant difference. Therefore, the *S. typhimurium* strain 22SD07 was selected for further investigation.

### 3.4. Infection with 22SD07 Significantly Reduces Body Weight and Affects Organ Indexes

**Figure 2:**
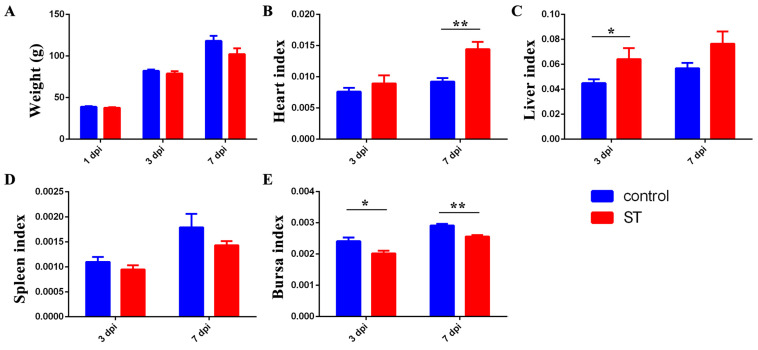
Effects of *S. typhimurium* infection on the body weight and organ indexes of ducks. (**A**) Effects of *S. typhimurium* infection on the body weight of ducks. (**B**–**E**) Effects of *S. typhimurium* infection on the heart, liver, spleen, and bursa indexes of ducks. Control: control group; ST: *S. typhimurium* infection group. Data are presented as mean ± SD. * *p* < 0.05, ** *p* < 0.01. dpi, days post-infection.

As shown in Figure 2, 22SD07 did not affect the body weight of the ducklings at 1 and 3 dpi, but the weight of the ducklings was reduced at 7 dpi (*p* > 0.05) (Figure 2A). Additionally, it significantly increased the heart index at 7 dpi and liver index at 3 dpi (Figure 2B,C), while it decreased the bursa index significantly at 3 and 7 dpi (Figure 2E).

### 3.5. S. typhimurium Translocation

**Figure 3:**
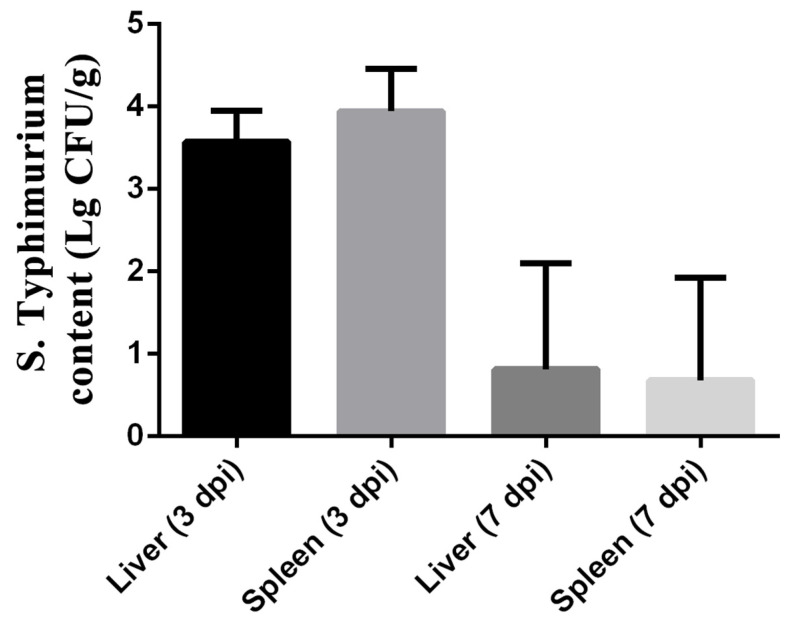
Colonization of *S. typhimurium* in liver and spleen of ST group on 3 and 7 dpi. The data are presented as the mean ± SD. dpi, days post-infection.

As shown in Figure 3, the colonization of *S. typhimurium* in the liver and spleen was observed by measuring the bacterial load of *S. typhimurium* at 3 and 7 dpi. The results showed the bacterial load of Salmonella in the liver reached 10^3.574±0.153^ cfu/g, while that in the spleen reached 10^3.942±0.2122^ cfu/g. However, the load of *S. typhimurium* in the liver and spleen was lower at 7 dpi than at 3 dpi. The bacterial load of Salmonella in the liver decreased to 10^0.8132±0.5267^ cfu/g, while that in the spleen decreased to 10^0.6799±0.5069^ cfu/g.

### 3.6. Histopathological Analysis

**Figure 4:**
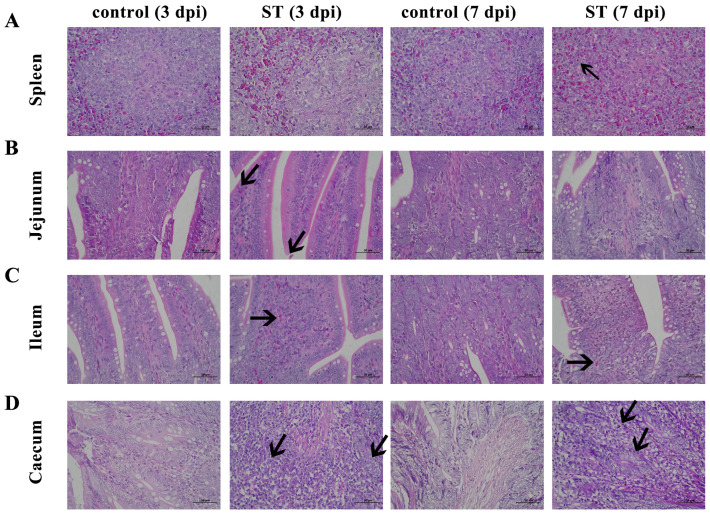
Histomorphology of spleen, jejunum, ileum, and cecum. Control: control group; ST: *S. typhimurium* infection group. (**A**) Poorly demarcated between white and red marrow, with punctate cellular necrosis (black arrows) in the spleen after *S. typhimurium* infection. (**B**) Connective tissue of the lamina propria is densely packed with erythrocytes visible in multiple capillaries in the jejunum after *S. typhimurium* infection. (**C**) Enterochromaffin epithelium is incomplete, with occasional detachment of the enterochromaffin epithelium and pitting necrosis in the ileum after *S. typhimurium* infection. (**D**) Large aggregates of lymphocytes (black arrows), shortening of the glandular ducts, a decrease in cells, and loose cytoplasm in the intestinal glandular epithelium in the cecum after *S. typhimurium* infection. dpi, days post-infection.

After the *S. typhimurium* infection, the splenic tissue is poorly demarcated between white and red marrow, with punctate cellular necrosis (black arrows), fragmented nuclei, and eosinophilic red staining of the cytoplasm (Figure 4A). The connective tissue of the lamina propria of the ileum is densely packed, with erythrocytes visible in multiple capillaries (Figure 4B). The jejunal enterochromaffin epithelium is incomplete, with occasional detachment of the enterochromaffin epithelium and pitting necrosis, cytoplasmic nuclear consolidation, and eosinophilic red staining of the cytoplasm (Figure 4C). In the cecum, there are large aggregates of lymphocytes (black arrows), a shortening of the glandular ducts, a decrease in the number of goblet cells, and loose cytoplasm in the intestinal glandular epithelium (Figure 4D).

### 3.7. Cytokine Expression

**Figure 5:**
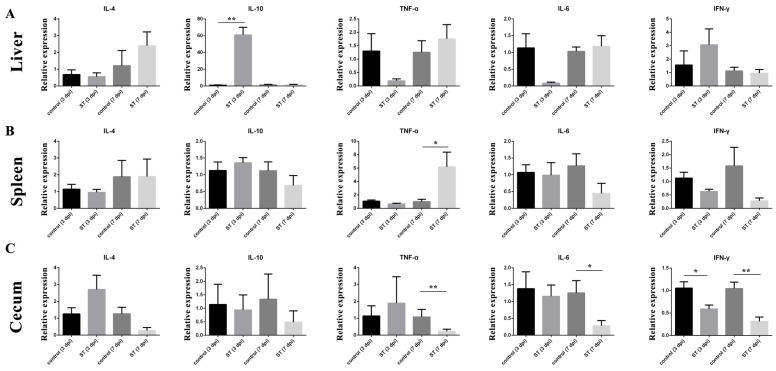
Effects of *S. typhimurium* infection on the expression of IL-4, IL-10, TNF-α, IL-6, and IFN-γ on 3 and 7 dpi. (**A**) Liver, (**B**) spleen, (**C**) cecum. control: control group; ST: *S. typhimurium* infection group. The data are presented as the mean ± SD. * *p* < 0.05, ** *p* < 0.01. dpi, days post-infection.

The relative expressions of cytokines in the liver, spleen, and cecum at 7 dpi were examined. The relative expression of IL-10 (*p* < 0.01) mRNA in the liver of the infected group was significantly higher than in the control at 3 dpi (Figure 5A), but the relative expression of TNF-α (*p* > 0.05) and IL-6 (*p* > 0.05) mRNA in the liver of the *S. typhimurium* group was lower than in the control (Figure 5B). In the spleen, the relative expression of TNF-α (*p* < 0.05) was significantly higher than in the control at 7 dpi. In the cecum, the relative expression of TNF-α (*p* < 0.01), IL-6 (*p* < 0.05), and IFN-γ (*p* < 0.01) mRNA in the cecum of the infected group was significantly higher than in the control at 3 dpi.

## 4. Discussion

The widespread and inappropriate use of antimicrobials has accelerated the emergence and spread of AMR [29]. Although infections caused by antimicrobial-resistant Salmonella have been reported worldwide, cases in developing countries are increasing significantly and at an alarming rate [30]. The clinical use of antibiotics is one of the main means of treating Salmonella disease, and with increasing farm density, the irrational use of antibiotics has led to the emergence of MDR strains. In this study, we examined 13,621 samples of duck cloacal swabs and dead embryos collected from duck farms located in Shandong Province between September 2022 and November 2023, and 44 strains of Salmonella were isolated and identified. The *S. typhimurium* isolates were then subjected to antimicrobial susceptibility testing to determine their resistance profile. The results showed that all *S. typhimurium* recovered from ducks had a high rate of resistance to DOX and AMK, which could be attributed to the overuse and misuse of tetracycline and aminoglycoside antibiotics in the treatment of duck diseases [31]. The rates of resistance to other antibiotics were not high, reflecting the fact that the amount of antibiotics used in duck meat production may be lower than in chicken farming. However, it cannot be ignored that indiscriminate use of antibiotics may increase the number of MDR Salmonella strains and the rate of antibiotic resistance. More importantly, the gradual increase in MDR Salmonella strains may cause public health problems [32].

All eight *S. typhimurium* strains carried *invA*, *sipC*, *sipA*, *ssaR*, *ssrA*, and *stnp1*. The major virulence factor of Salmonella is *invA*, which encodes a protein that makes Salmonella highly pathogenic. Many studies have shown that there is a correlation between the virulence genes contained in Salmonella and their own virulence, mainly due to the interaction between a large number of virulence genes [33,34]. During Salmonella invasion, the bacterial effectors *SipC*, *SopE2,* and *SopB* recruit exocyst subunits from membrane reservoirs and other cellular compartments, thereby allowing exocyst complex assembly and membrane delivery required for bacterial uptake [35]. Both the T3SS translocator *SipC* and effector *SipA* are critical for Salmonella infection by subversion of the host cell cytoskeleton, but the precise molecular interplay between them remains unknown. It has been shown that *SipA* binds along the F-actin grooves with a unique binding pattern using cryoelectronic microscopy. *SipA* stabilizes F-actin through charged interface residues and appears to prevent inorganic phosphate release through the closure of the “back door” of the adenosine 5′-triphosphate pocket. Moreover, *SipC* promotes the binding of *SipA* to F-actin [36]. In addition, a variety of virulence genes are clustered on the so-called Salmonella Pathogenicity Islands (SPIs); SPI-2 presents the *ssaR* and *sifA* genes, which are necessary for the survival and subsequent multiplication of the bacterium and also play an important role in systemic infection [37,38]. The *SsrAB* system is encoded by the *ssrAB* operon located in SPI-2. *SsrA* is the sensor kinase, and *SsrB* is the response regulator that directly controls the expression of target genes [39]. The *SsrAB* two-component system is the central positive regulator for the SPI-2 genes and other functionally related genes located outside SPI-2 [40,41].

Biofilm formation is a key strategy for the survival of Salmonella under unfavorable environmental conditions [42]. Some bacterial cell surface components, such as cellulose, flagella, and hyphae, have been shown to contribute to the attachment of Salmonella to different surfaces [43]. In this study, the *S. typhimurium* strain known as 22SD07 was highly biofilm-forming, and drug susceptibility tests showed it to be MDR. So, we further explore its pathogenicity to ducklings. An injection dose of 10^8^ CFU *S. typhimurium* (0.32 LD_50_) was selected by leg muscle injection, and diseased ducks with obvious clinical characteristics were obtained from this infection experiment in Cherry Valley broiler ducklings. The results showed that the bacterial content of the organs had decreased significantly but was still present at 7 dpi (Figure 3). It has been shown that the number of heterophilic cells in the spleen is significantly increased in susceptible chicken breeds infected with Salmonella [44,45]. Salmonella uses virulence factors to gain entry into the intestinal epithelium and survive in mucosal macrophages, leading to an acute inflammatory process [46]. Inflammatory bowel disease is an idiopathic inflammatory disease of the intestines affecting the ileum, rectum, and colon. The main manifestations are diarrhea, abdominal pain, and bloody stools. The pathological changes induced by Salmonella include enteritis and septicemias. The intestinal mucosa displays punctate hemorrhage, thickening of the cecum wall, intestinal mucosa congestion, enlargement of the mesenteric lymph node, necrosis, and other characteristics. It has been demonstrated that infection with avian *S. typhimurium* increases mortality and intestinal diseases and leads to gastroenteritis related to human food-borne diseases [45]. The pathological section results of this experiment showed notable inflammatory alterations and tissue damage. The most significant changes were observed in the spleen, which exhibited an indistinct boundary between white and red medulla, infiltration of lymphocytes, and cytoplasmic eosinophilic red staining (Figure 4A). Hemorrhages were observed in the jejunum, ileum, and spleen, accompanied by connective tissue hyperplasia and cell infiltration (Figure 4B–D). Additionally, cellular necrosis was evident in the jejunum and spleen. The mucosal barrier is destroyed by inducing desquamation of the epithelium, the loss of mucin-producing goblet cells, and the overall distorted villi shape (becoming shorter and wider), as findings have been reported previously [47,48]. This mucosal destruction and inflammation lead to the interference of intestinal function, promote the progress of *Salmonella typhimurium* colonization, and cause a loss of weight gain [49,50].

## 5. Conclusions

The isolation rate of *S. typhimurium* in some duck farms in Shandong Province was 0.06% (8/13,621), and MDR *S. typhimurium* accounted for 37.5% (3/8), all carrying six kinds of virulence genes, including *invA*, *rck*, *sipC*, *pefA*, *sipA,* and *ssaR*. *S. typhimurium* 22SD07 caused decreased body weight, increased organ index, and intestinal villous shedding.

## Figures and Tables

**Table 1:** List and sequences of primers used in this study.

Primer	Sequence	Genebank
DuIFN-γ-F	GCTGATGGCAATCCTGTTTT	KF746069.1
DuIFN-γ-R	GGATTTTCAAGCCAGTCAGC	
DuIL-10-F	GGGGAGAGGAAACTGAGAGATG	NM_001310368.1
DuIL-10-R	TCACTGGAGGGTAAAATGCAGA	
DuIL-6-F	TTCGACGAGGAGAAATGCTT	JF437643.1
DuIL-6-R	CCTTATCGTCGTTGCCAGAT	
DuTNF-α-F	ATCAGCTGGCTAAGACCGTG	XM_005506221.3
DuTNF-α-R	GGGATTGTACAAGGCAGCCA	
DuIL-4-F	ATCCTCTCCACGCAGGTTTC	MF346730.1
DuIL-4-R	TGGTGCTCTTTGTCACGATG	
18S-F	TCAGATACCGTCGTAGTTCC	
18S-R	TTCCGTCAATTCCTTTAAGTT	

**Table 2:** Drug resistance profiles and virulence genes of *S. typhimurium* strains.

No.	Serotype	Drug Resistance	Virulence Genes
22SD01	*S. typhimurium*	AMK-TMP	*invA*, *sipC*, *sipA*, *ssaR*, *ssrA*, *stnp1*, *hilA*
22SD02	*S. typhimurium*	AMK-TE-DOX	*invA*, *sipC*, *sipA*, *ssaR*, *ssrA*, *stnp1*, *hilA*
22SD03	*S. typhimurium*	AMK	*invA*, *sipC*, *sipA*, *ssaR*, *ssrA*, *stnp1*, *hilA*
22SD04	*S. typhimurium*	AMK-DOX	*invA*, *sipC*, *sipA*, *ssaR*, *ssrA*, *stnp1*
22SD05	*S. typhimurium*	DOX	*invA*, *sipC*, *sipA*, *ssaR*, *ssrA*, *stnp1*, *hilA*
22SD06	*S. typhimurium*	DOX	*invA*, *sipC*, *sipA*, *ssaR*, *ssrA*, *stnp1*, *hilA*
22SD07	*S. typhimurium*	CTX-DOX-CTR-TE-AMX-AMP-CAZ	*invA*, *sipC*, *sipA*, *ssaR*, *ssrA*, *stnp1*, *hilA*
22SD08	*S. typhimurium*	AMK-DOX-ENR	*invA*, *sipC*, *sipA*, *ssaR*, *ssrA*, *stnp1*, *hilA*

Abbreviation: amikacin (AMK); trimethoprim (TMP); tetracycline (TE); doxycycline (DOX), (CTX); ceftriaxone (CTR); amoxicillin (AMX); ampicillin (AMP); ceftazidime (CAZ); enrofloxacin (ENR).

**Table 3:** The proportion of drug resistance of *S. typhimurium* strains.

Drug Resistance	Proportion (%)
DOX	75 (6/8)
AMK	62.5 (5/8)
TE	25 (2/8)
TMP	12.5 (1/8)
CTX	12.5 (1/8)
CTR	12.5 (1/8)
AMX	12.5 (1/8)
AMP	12.5 (1/8)
CAZ	12.5 (1/8)
ENR	12.5 (1/8)

## Data Availability

The original contributions presented in the study are included in the article; further inquiries can be directed to the corresponding authors.
